# Identification of P2Y receptors involved in oleamide-suppressing inflammatory responses in murine microglia and human dendritic cells

**DOI:** 10.1038/s41598-019-40008-8

**Published:** 2019-02-28

**Authors:** Masahiro Kita, Yasuhisa Ano, Asuka Inoue, Junken Aoki

**Affiliations:** 1 Research Laboratories for Health Science & Food Technologies, Kirin Company Ltd, 1-13-5 Fukuura Kanazawa-ku, Yokohama-shi, Kanagawa 236-0004 Japan; 2 Graduate School of Pharmaceutical Sciences, Tohoku University, 6-3, Aoba, Aramaki, Aoba-ku, Sendai City, Miyagi 980-8578 Japan

## Abstract

Microglia, a type of immune cell in the central nervous system, are involved in inflammation leading to neurodegenerative diseases. We previously identified oleamide from fermented dairy products as a neuroprotective compound suppressing microglial inflammation. Oleamide is an endocannabinoid and displays anti-inflammatory activity via the cannabinoid-2 (CB2) receptor; however, the mechanism underlying this anti-inflammatory activity has not been fully elucidated. Here, we found that the suppressive effect of oleamide on microglial tumor necrosis factor-α (TNF-α) production was canceled by inhibitors of G-protein-coupled receptor (GPCR) downstream signaling but not by a CB2 antagonist, suggesting that GPCRs other than CB2 are involved in the anti-inflammatory effects of oleamide. An extensive screen for GPCRs using a transforming growth factor-α shedding assay system identified P2Y1, P2Y4, P2Y6, P2Y10, and P2Y11 as candidates for the oleamide target. P2Y1 and P2Y10 agonists suppressed microglial TNF-α production, while a pan P2 receptor antagonist canceled the suppressive effect. Furthermore, we observed a relationship between the P2Y1 agonistic activities and the suppressive activities of oleamide and its analogs. Taken together, our results suggest that, in addition to CB2, P2Y type receptors are the potential targets of oleamide, and P2Y1 plays a role in the suppression of microglial inflammatory responses by oleamide. (200/200 words)

## Introduction

In neurodegenerative disorders, such as Alzheimer’s disease, Parkinson’s disease, and depression, chronic inflammation featuring excessive activation of microglia is closely involved in the pathogenesis^1–3^. Microglia are a specialized population of macrophages in the central nervous system (CNS) that maintain the CNS environment by removing apoptotic cells and by regulating synaptic plasticity and synaptic pruning^4^. However, an accumulation of amyloid-β and chronic psychological stress result in overactivation of microglia, which release an excess of inflammatory cytokines including TNF-α and reactive oxygen species (ROS), leading to neuronal cell death and neurodegenerative disease. Indeed, elevated levels of TNF-α in the CNS have been reported to promote the pathology of neurodegenerative disease^5^. Thus, regulation of microglial activation is thought to be an attractive strategy for treatment and prevention of these disorders.

We have previously identified oleamide (cis-9-octadecanamide, OAD) as a compound in fermented dairy products such as Camembert cheese that is responsible for suppressing microglial inflammatory responses^6^. OAD, which is synthesized from oleic acid by *Penicillium candidum* during fermentation, suppresses microglial production of TNF-α in response to lipopolysaccharide (LPS) stimulation, as well as the expression of inflammatory cell markers, *in vitro* and *in vivo*. OAD was first reported as an agonist of cannabinoid receptors (CB1 and CB2) and is well known to induce sleep^7,8^.

CB1 is expressed on neurons of the CNS, and CB2 is expressed mainly on immune cells including microglia^9^. It is well established that microglial activation of CB2 suppresses the release of proinflammatory molecules *in vitro* and *in vivo*, and thereby leads to neuroprotection^9^. *In vitro*, treatment with cannabinoids suppresses microglial TNF-α production in response to LPS stimulation^10^, and some of the anti-inflammatory effects of OAD are dependent on CB2 activation^11^. However, treatment with antagonists of CB2 does not completely attenuate the anti-inflammatory activity of OAD, and OAD displays a synergistic effect with other CB2 agonists^11^. These reports have encouraged us to explore potential receptors for OAD other than CB2.

In the present study, we explored whether G-protein-coupled receptors (GPCRs) play a role in the anti-inflammatory activity of OAD, because GPCR signaling inhibitory assays suggested that multiple GPCRs might be involved in OAD’s anti-inflammatory activity. A series of GPCR screens were conducted by using a transforming growth factor-α (TGF-α) shedding assay^12^, and the identified GPCRs were evaluated for their involvement in the anti-inflammatory effects of OAD in primary murine microglia and human dendritic cells (DCs).

## Results

### OAD partially suppresses TNF-α production by murine microglia via the CB2 receptor

**Figure 1:**
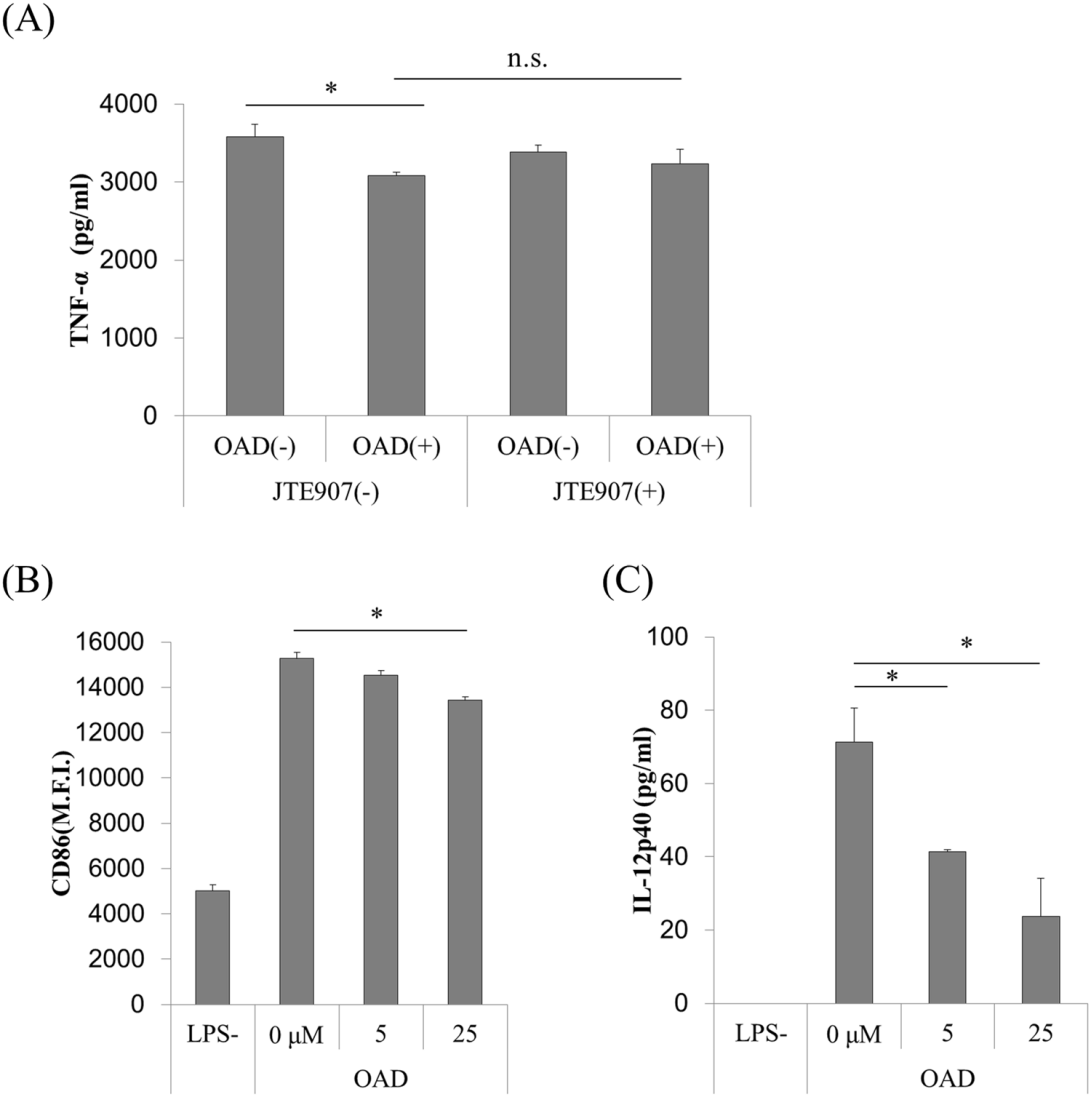
The effects of OAD and JTE907 on inflammatory responses in murine microglia and human DCs. (**A**) Primary murine microglia were treated with 100 ng/ml of JTE907 for 1 h, followed by 5 μM OAD overnight. They were then stimulated by 5 ng/ml of LPS and 0.5 ng/ml of IFN-γ for 12 h. Extracellular TNF-α was measured by ELISA. (**B**,**C**) Human DCs were treated with OAD for 24 h, followed by 1 μg/ml of LPS, and 100 ng/ml of IFN-γ for 24 h. The expression of CD86 was then measured by FACS (**B**), and extracellular IL-12p40 was measured by ELISA (**C**). The data represents mean ± S.E.M. (n = 3) (*shows p < 0.05 tested by Tukey–Kramer’s test).

To investigate the involvement of CB2 in the suppressive effects of OAD on microglial TNF-α production, primary murine microglia were treated with JTE907, a CB2-selective inverse agonist, before LPS treatment. Less suppression of microglial TNF-α production by OAD was observed for cells treated with JTE907 (Fig. 1A), showing that CB2 is involved in the suppression of TNF-α by OAD; however, the difference between cells with and without JTE907 (columns 2 and 4 in Fig. 1A) did not reach significance; in other words, treatment with JTE907 did not completely attenuate the effect of OAD on microglia. These results suggest that receptors other than CB2 might be involved in the mechanism underlying OAD’s suppression of microglial TNF-α production.

### OAD suppresses the expression of inflammatory markers and cytokines in human DCs

To elucidate the anti-inflammatory activity of OAD in human monocytes including DCs and microglia, human DCs differentiated from peripheral blood mononuclear cells (PBMCs) were treated with OAD followed by LPS stimulation. OAD dose-dependently suppressed both the expression of CD86 on human DCs, as measured by flow cytometry (Fig. 1B), and the production of interleukin-12p40 (IL-12p40) (Fig. 1C). Thus, OAD suppresses the inflammatory response of human monocytes as well as murine cells.

### Inhibition of downstream signaling of GPCRs attenuates the suppression of microglial TNF-α production by OAD

**Figure 2:**
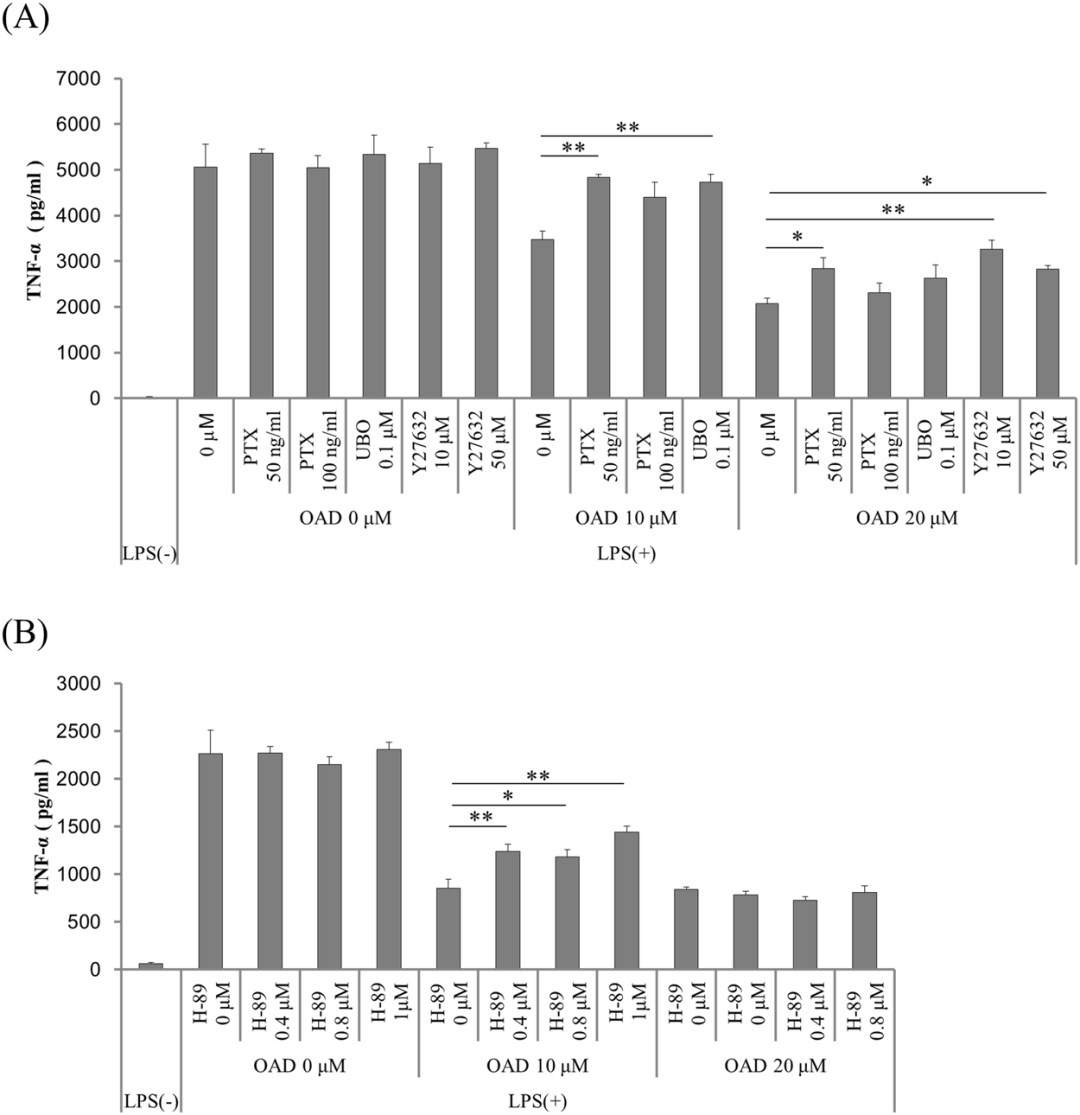
The effects of inhibitors of GPCR downstream signaling on the suppression of TNF-α production by OAD in murine microglia. (**A**,**B**) Primary mice microglia were treated with 50 or 100 ng/ml of PTX for 6 h (**A**), 0.1 μM UBO-QIC for 1 h (**A**), 10 or 50 μM Y27632 (**A**), or 0.4, 0.8, or 1 μM H-89 (Gs signal inhibitor) for 1 h (**B**), followed by OAD treatment overnight. They were then stimulated by 5 ng/ml of LPS and 0.5 ng/ml of IFN-γ for 12 h. Extracellular TNF-α was measured by ELISA. The data represents mean ± S.E.M. (A, n = 3; B, n = 5). (*p < 0.05, **p < 0.01 vs 0 μM control by Dunnett’s test).

To identify receptors other than CB2 that might be involved in the anti-inflammatory effects of OAD, the potential contribution of GPCRs was examined by using inhibitors of their downstream signaling. After pretreatment with inhibitors of Gi (pertussis toxin, PTX), Gq (UBO-QIC), G_12/13_ (Y-27632), and Gs (H-89) downstream signaling, microglia were treated with OAD and then stimulated by LPS and interferon-γ (IFN-γ). The suppression of TNF-α production by OAD was significantly attenuated by pretreatment with PTX, an inhibitor of the main downstream signaling pathway of CB2 (Fig. 2A). Pretreatment with other GPCR downstream inhibitors (UBO-QIC, Y-27632 and H-89) also attenuated the effects of OAD (Fig. 2A,B). Thus, these results suggest that OAD has potential agonistic activities on GPCRs other than CB2 and mediates its activity to suppress TNF-α production through these receptors.

### P2Y1, P2Y4, P2Y6, P2Y10, and P2Y11 are potential receptors for OAD

**Figure 3:**
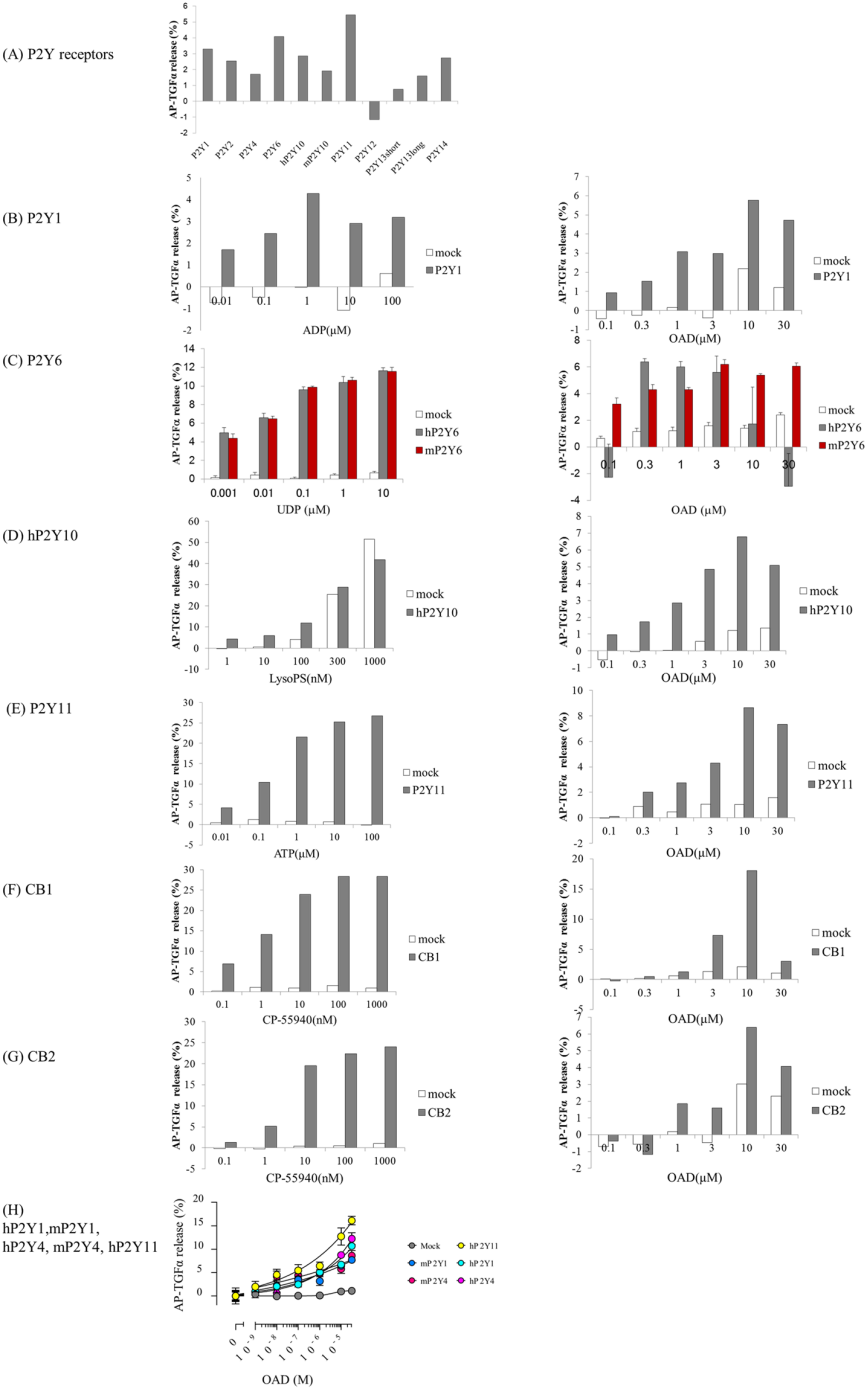
Improvement in the TGF-α shedding response to OAD in HEK293 cells expressing P2Y1, P2Y4, P2Y6, P2Y10, P2Y11, CB1, and CB2. (**A**) Summary of the results of the TGF-α shedding response in cells transfected with P2Y receptors during the screening series. Mock transfected cells (pCAGGS empty vector) (white bar) (**B**–**H**) or HEK 293 cells expressing GPCRs (left) (P2Y1 (**B**,**H**), human P2Y6 (**C**, dark bar), mouse P2Y6 (**B**, red bar), P2Y10 (**D**), P2Y11 (**E**,**H**), CB1 (**F**), CB2 (**G**), and human and mouse P2Y4 (**H**)) were treated with OAD (right) or each receptor agonist (left) (ADP (**B**), UDP (**C**), LysoPS (**D**), ATP (**E**), CP-55940 (**F**,**G**)). The TGF-α shedding response was then calculated. Data represent the mean (n = 2) (**A**,**B**, **D**–**F**), mean ± S.E.M (n = 4) (**C**), or mean ± S.E.M (n = 3) (**H**).

The above inhibition assays of GPCR downstream signaling demonstrated the involvement of other GPCRs in OAD’s suppression of microglial TNF-α production, although no GPCR receptors for OAD other than CB1 and CB2 have been previously reported. Thus, to identify novel receptors for OAD, we screened 543 GPCRs using a TGF-α shedding assay system^12^. Supplementary Table 1 presents the results of TGF-α shedding responses from cells transfected with each GPCR in the presence of 20 μM OAD, a concentration that displays anti-inflammatory activity in human monocytes and murine microglia. Figure 3A shows the TGF-α shedding responses from cells transfected with each P2Y receptor. Receptors indicating agonistic activity of OAD were further tested in the presence of 0.1–30 μM OAD.

This screening series revealed that OAD increased the concentration of TGF-α in the supernatant in a dose-dependent manner in human embryonic kidney (HEK) cells transfected with human P2Y1, P2Y4, P2Y6, P2Y10, P2Y11, CB1, and CB2; in addition, TGF-α levels were higher than in HEK cells transfected with mock plasmid (Fig. 3B–H). TGF-α shedding responses were also increased in cells expressing murine P2Y1, P2Y4, and P2Y6, and the responses of the murine receptors were comparable to those of the human receptors (Fig. 3C,H). Furthermore, the agonistic activity of OAD against human P2Y1 was detected in another assay based on the β-arrestin recruitment system (data not shown). Collectively, these results suggest that P2Y1, P2Y4, P2Y6, P2Y10, and P2Y11, in addition to CB1 and CB2, are potential targets for OAD.

### P2Y1 and P2Y10 agonists suppress microglial TNF-α production, and a P2 receptor antagonist attenuates the suppressive effects of OAD

**Figure 4:**
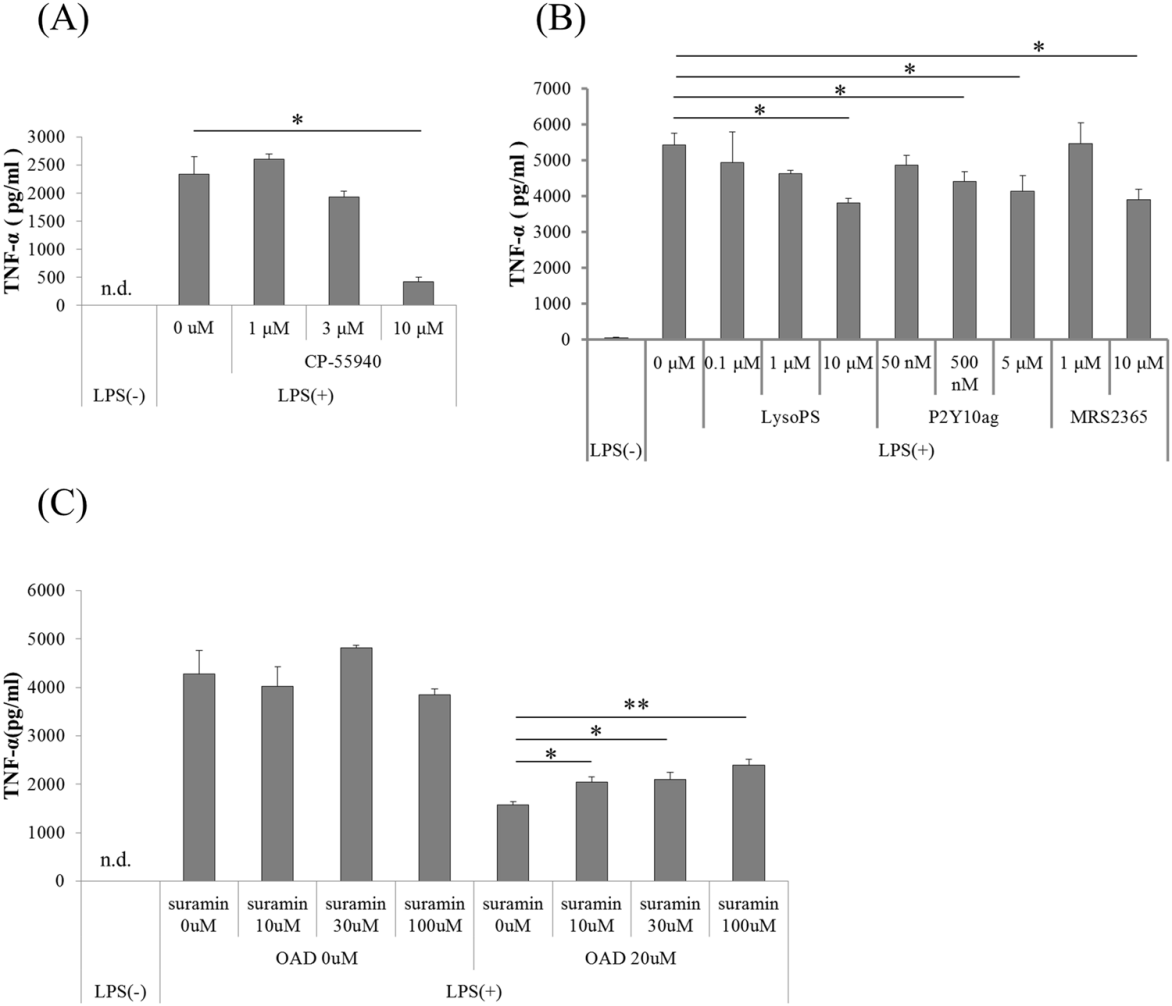
The effects of GPCR agonists and antagonist on microglial TNF-α production. (**A**,**B**) Primary mice microglia were treated with CP-55940 (**A**), or LysoPS, a P2Y10-selective agonist, or MRS2365 (**B**) overnight. The data represent mean ± S.E.M (n = 3–6). (*p < 0.05 vs 0 μM control by Williams’s test in each agonist group.) (**C**) Cells were treated with different concentrations of suramin for 30 min, followed by 20 μM OAD. Data represents mean ± S.E.M. (n = 3). (*p < 0.05, **p < 0.01 vs 0 μM control by two-tail Dunnett’s test).

To investigate the specific involvement of the above-identified GPCRs in the microglial inflammatory response, microglia were treated with a selective agonist of each GPCR before LPS stimulation, and the production of TNF-α was evaluated. Consistent with previous results, CP-55940 (CB1/CB2 agonist) significantly suppressed the LPS-induced microglial production of TNF-α (Fig. 4A)^10^. Moreover, treatment with MRS2365 (P2Y1-selective agonist) and lysophosphatidylserine (LysoPS, P2Y10 agonist) and a P2Y10-selective agonist also dose-dependently suppressed microglial TNF-α production (Fig. 4B). On the other hand, MRS2957 (P2Y6-selective agonist) did not suppress TNF-α production (data not shown).

Next, to investigate whether the suppressive effect of OAD on TNF-α production is dependent on these GPCRs, the effect of suramin, a non-selective P2 receptor antagonist, on microglial TNF-α production was investigated. Previous studies showed that 10–100 μM suramin inhibited the response of P2Y1 receptor to 2-methyl adenosine triphosphate^13^. In addition, we confirmed that 10–100 μM suramin attenuated the suppression of microglial TNF-α production by ATP (data not shown); therefore, we employed a concentration of 10–100 μM suramin for this investigation. Pretreatment of suramin significantly attenuated the OAD-mediated suppression of microglial TNF-α production (Fig. 4C). Collectively, these results suggests that activation of P2Y1 and P2Y10 suppresses the microglial production of TNF-α in response to LPS stimulation, and P2 receptors are involved in the mechanism of OAD suppression of TNF-α production.

### OAD analogs displaying high agonistic activity against P2 receptors more strongly suppress microglial TNF-α production

**Figure 5:**
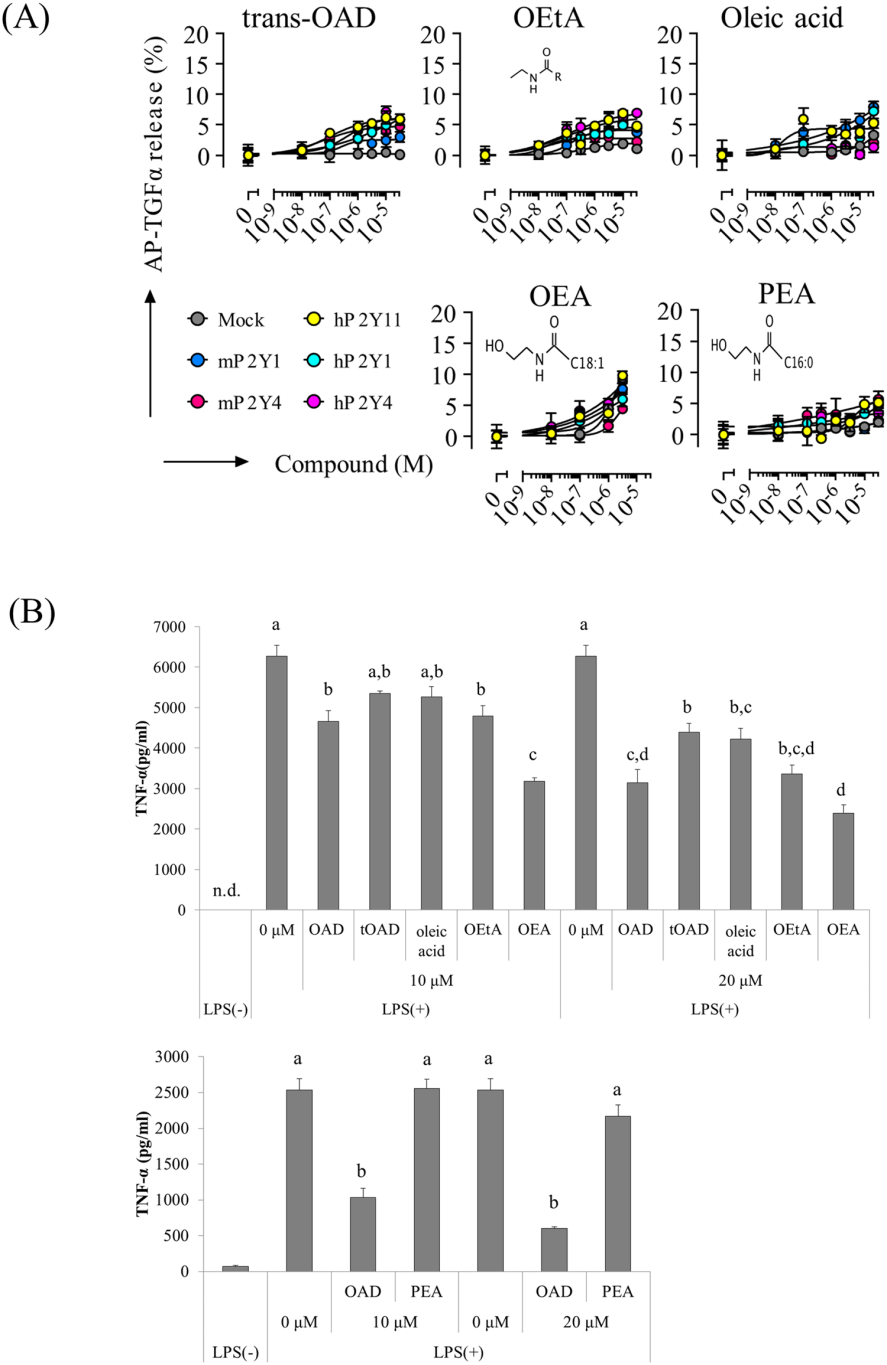
Comparison of agonistic activity and TNF-α suppressing activity between OAD and its analogs. (**A**) Cells expressing human and murine P2Y1 and P2Y4, and human P2Y11 were treated with OAD, tOAD, oleic acid, OEtA, PEA, or OEA, and AP-TGFα release was measured. (**B**) Primary microglia were treated with OAD and its analog derived from oleic acid (**B**, upper), or PEA (**B**, lower) overnight, followed by LPS stimulation. Bars with a different letter were statistically different by Turkey–Kramer’s test. Statistical analysis was performed between a control 0 μM value and each concentration. The data represent mean ± S.E.M (n = 3).

To investigate further the relationship between P2 agonistic activity and the suppression of TNF-α production, the activity of several OAD analogs was investigated. Trans-OAD (tOAD), oleoylethylamide(OEtA), oleic acid, and palmitoylethanolamide (PEA) displayed lower agonistic activity against P2Y1, P2Y4, and P2Y11 as compared with OAD, whereas oleoylethanolamide (OEA) had agonist activity comparable to that of OAD (Figs 3H and 5A). In addition, the TNF-α-suppressing activity of tOAD, oleic acid, and PEA was lower than that of OAD, whereas the activity of OEA and OEtA was comparable to that of OAD (Fig. 5B). Collectively, these results suggest that high agonistic activity against these GPCRs leads to high suppression of microglial TNF-α production.

## Discussion

Our group previously demonstrated that OAD obtained from Camembert cheese displays preventive effects on Alzheimer’s disease by suppressing the microglial inflammatory response *in vitro* and *in vivo*. OAD suppresses the production of microglial inflammatory cytokines in response to LPS stimulation and induces microglia to differentiate into an M2 anti-inflammatory type. OAD is well known as a CB1 and CB2 agonist, and these activities of OAD are partially dependent on CB2, although the involvement of other mechanisms has been suggested. In the present study, an extensive GPCR screening assay identified P2Y receptors as novel receptors for OAD that are involved in its anti-inflammatory activity. Among the P2Y receptors, P2Y1 and P2Y11 are particularly involved in the activity of OAD in murine microglia and human DCs, respectively.

CB2 is primarily expressed in immune cells, including monocytes and microglia^9^, and its activation contributes to the suppression of microglial activation *in vivo* and *in vitro*^10,14,15^. Both CB1 and CB2 are expressed in quiescent microglia, but CB2 is abundant in inflammatory microglia^16^. Furthermore, several studies have suggested that the suppression of microglial inflammatory responses by cannabinoids is attenuated by CB2 blockade, but not by CB1 blockade^16,17^. Thus, it is considered that cannabinoids suppress microglial activation mainly via CB2. A previous study has shown that the suppression of microglial inflammatory response by OAD is partially, but not fully, attenuated by treatment with a CB2-selective antagonist (AM 630), and that OAD displays a synergistic effect with other CB2 agonists^11^, suggesting that OAD suppresses microglial inflammatory responses via CB2 either indirectly or via other receptors. Therefore, we employed a CB2-selective inverse agonist, JTE907 (EC50 for mouse CB2, 22 nM^18^) to block the indirect effects of OAD. We used a concentration of 100 ng/ml (228 nM) JTE907, which is sufficient to work as an inverse agonist for CB2. We found, however, that JTE907 could not fully attenuate OAD’s suppression of microglial TNF-α production. Collectively, these data suggest that GPCRs other than CB2 are involved in the anti-inflammatory effects of OAD on microglia.

To identify receptors for OAD, we used a recently developed TGF-α shedding assay^12^, which is based on GPCR-activated induction of ectodomain shedding of alkaline phosphatase-tagged TGF-α (AP-TGF-α) and can detect multiple GPCR downstream signaling pathways via the use of chimeric Gα proteins. This assay has the potential to detect the activation of almost all known ligand and orphan GPCRs, and thus was considered suitable for exploring the stimulation of GPCRs by OAD.

In this study, P2Y1 was identified as a novel target for OAD. P2Y1 is a member of the P2Y purinergic metabotropic receptor subfamily. It is expressed in neurons, astrocytes, and microglia, and is activated by ATP and ADP. The inflammatory response mediated by P2Y1 in the CNS is induced by extracellular ATP released from cells that have died owing to ischemia or brain damage; in addition, it has also been reported that blockade of P2Y1 modulates brain damage^19,20^. Both protective and detrimental roles of astrocytic P2Y1 in brain pathology have been reported in a mouse model of ischemia^19,21,22^ and a neuron–glia co-culture model^23^. On the other hand, the activation of neuronal P2Y1 disrupts circuit excitability and might induce neuronal excitotoxicity^24^. As compared with astrocytes and neurons, there are few reports on the role of P2Y1 in microglia.

In primary microglia and in microglial cell lines, high concentrations of extracellular ATP (>1 mM) activate P2X7, the purinergic ionotropic receptor, and promote the production of pro-inflammatory molecules including nitric oxide, interleukin-6 (IL-6), interleukin-1β, and TNF-α^25–29^. By contrast, a low concentration of ATP (<300 μM) activates P2Y receptors and suppresses the production of pro-inflammatory cytokines^30,31^. The P2Y family agonist 2MeSATP suppresses the production of pro-inflammatory cytokines, whereas the P2X7 agonist BzATP promotes this production^30^. The present study confirmed that the P2Y1 agonist MRS2365 suppresses the production of TNF-α in response to LPS stimulation. Furthermore, OAD analogs showing high agonistic activity against P2Y1 more strongly suppressed TNF-α production. The OAD-mediated suppression of TNF-α production was attenuated by UBO-QIC, a Gq signaling inhibitor, and suramin, a P2 receptor antagonist. Collectively, these results suggest that the activation of P2Y1 is involved in the anti-inflammatory effect of OAD on microglia.

P2Y11 is not found in the murine genome^32^ and is expressed on human immune cells such as DCs^32^. In murine cells, the role of P2Y11 might be compensated by P2Y1, which shares greatest homology with P2Y11^33^. The present study showed that OAD also suppressed the production of both IL12p40, a pro-inflammatory cytokine, and CD86, a costimulatory molecule, in human DCs. These cells are antigen-presenting cells that play key roles in the regulation of immune responses. The endocannabinoid system including CB2 in DCs is involved in the suppression of inflammatory response^34^. It has also been reported that, upon activation by ATP, P2Y11 suppresses the inflammatory response in DCs^35,36^. Extracellular ATP induces DCs to differentiate into an anti-inflammatory type and suppresses the secretion of pro-inflammatory cytokines, including IL-12p70 and IL-6 via P2Y11;^36^ in addition, NF546, a P2Y11-selective agonist, suppresses IL-12p70 production^35^. Taken together, these observations indicate that CB2 and P2Y11 on DCs are potentially involved in the anti-inflammatory effects of OAD. Further studies using human DCs lacking P2Y11 are needed to evaluate fully the role of P2Y11 in the effects of OAD.

In the present study, we also identified P2Y4, P2Y6, and P2Y10, which are expressed on microglia, as OAD targets. P2Y4 is activated by UTP and ATP, and P2Y6 is activated by UDP. It has been reported that P2Y4 is involved in pinocytosis induced by ATP^37^, and P2Y6 is involved in phagocytosis induced by UDP^38^ in microglia. To our knowledge, however, there are no reports on the involvement of these receptors in modulating the production of inflammatory molecules. On the other hand, P2Y10 coupling with G_12/13_ is activated by LysoPS, and it has been previously reported that LysoPS modulates microglial morphology and suppresses the microglial inflammatory response^39^. Further studies are necessary to clarify the physiological and pathological roles of stimulation of these receptors by OAD.

In addition to P2Y receptors, other GPCRs such as G2A and EP3 were identified are possible targets of OAD by a series of screens. However, we did not examine these receptors in detail in the present study. Further research will elucidate whether or not OAD activates these receptors, and, if so, the physiological effects of stimulation of these receptors by OAD.

In conclusion, this is first report to identify P2Y1, P2Y4, P2Y6, P2Y10 and P2Y11 as novel targets for OAD. Our findings also suggest that P2Y1 and P2Y11 are involved in the anti-inflammatory activity of OAD in murine microglia and human DCs, respectively.

## Materials and Methods

### Animals

All animal care and experimental procedures were performed in accordance with the guidelines of the Committee for Animal Experimentation at Kirin Company, Ltd. The studies were approved by the Committee for Animal Experimentation at Kirin Company, Ltd (approval No; AN10108-Z00, AN10109-Z00, AN10110-Z00, AN10173-Z00 and AN10315-Z00). For the assay using primary microglia, pregnant C57BL6/J mice were purchased from Charles River Japan (Tokyo, Japan) and kept in a room maintained on a 12-h/12-h light/dark cycle at a temperature of 23 ± 1 °C with free access to standard mouse chow (CE-2; CLEA Japan, Tokyo, Japan) and water. All efforts were made to minimize animal suffering.

### Primary microglia cell culture

Brain cells were obtained from new born mice (<2-week-old) using a Neural Tissue Dissociation Kit (P) (Milteny Biotec, Cologne, Germany), treated with CD11b antibody-conjugated microbeads (Milteny Biotec), and then isolated by magnetic cell sorting as CD11b^+^ cells, as previously described^6^. The isolated cells were plated on poly-D-Lysine (PDL)-coated 96-well plates (BD Biosciences, CA, USA), and then cultured in DMEM/F12 (Gibco, CA, USA) with 10% fetal bovine serum (FBS; Gibco) containing 100 U/ml of penicillin/streptomycin (Sigma-Aldrich, MO, USA).

### *In vitro* microglial TNF-α production assay

Isolated microglia were plated at density of 3 × 10^4^ cells per well on PDL-coated plates. For the assays using inhibitors or antagonists, microglia were pretreated with H-89 (protein kinase A, downstream inhibitor of Gαs; Sigma-Aldrich), UBO-QIC (Gq inhibitor)^40^, Y-27632 (Rho-associated coiled-coil-forming kinase; downstream inhibitor of Gα_12/13_; Sigma-Aldrich), and JTE907 (CB2-selective antagonist; TOCRIS, Bristol, UK) for 1 hour; with PTX (Gi inhibitor; Wako, Tokyo, Japan) for 6 hours; or with suramin (P2 receptor antagonist; Sigma-Aldrich) for 30 min. After pretreatment, microglia were treated with OAD (Sigma-Aldrich) overnight, and then stimulated with 5 ng/ml of LPS (Sigma-Aldrich) and 0.5 ng/ml of IFN-γ (R&D Systems, MN, USA) for 12 hours. CP-55940 (Sigma-Aldrich), MRS2365 (TOCRIS), LysoPS (P2Y10 agonist^12^), P2Y10-selective agonist^41^ or OAD analogs, including tOAD (Toronto Research Chemicals, Toronto, Canada), oleic acid (Wako, Osaka, Japan), OEtA (Cayman Chemical, MI, USA), OEA (Cayman Chemical), and PEA (Cayman Chemical), were tested as agonists for each GPCR. The concentration of TNF-α in the culture supernatant was quantified by enzyme-linked immunosorbent assay (ELISA) using Mouse TNF-α ELISA Ready-SET-Go (eBioscience, CA, USA).

### Generation and culture of human PBMC-derived DCs

CD14^+^ cells were isolated from human PBMCs (Lonza Japan, Tokyo, Japan) using a Monocyte Isolation kit (Milteny Biotec). Cells were cultured for 6 days in RPMI 1640 medium containing 10% FBS (Invitrogen, CA, USA), 50 U/ml of penicillin, 50 μg/ml of streptomycin (Invitrogen), 50 μM 2-mercaptoethanol (Invitrogen), 50 ng/ml of granulocyte macrophage colony-stimulating factor (GM-CSF, R&D systems), and 20 ng/ml of interleukin-4 (IL-4, R&D systems). On day 6 of culture, cells were re-suspended and plated at density of 1.2 × 10^5^ cells per well. Cells were treated with OAD for 24 hours. Next, cells were stimulated with 1 μg/ml of LPS (Sigma-Aldrich) and 100 ng/ml of IFN-γ (R&D Systems) for 24 hours. The supernatant was then collected for the quantification of IL-12p40, and the cells were collected for flow cytometry. The amount of IL-12p40 in the supernatant was measured by ELISA using a Mouse IL-12p40 ELISA kit (BD Pharmingen). Cells were stained with anti-CD86-PE (eBioscience, CA, USA), anti-CD11c-PE-Cy7 (BD Pharmingen), anti-HLA-DR-PerCP (BD Bioscience), and 7AAD (BD Bioscience), and the expression of CD86 and I-A/I-E in CD11c^+^ and 7AAD^−^ cells was assessed. This experiment was done in accordance with the guidelines of Kirin Company, Ltd and approved by the clinical research ethics committee of Kirin Company, Ltd.

### TGF-α shedding assay

HEK 293 A cells were maintained in DMEM containing 10% FCS, penicillin, streptomycin, and glutamine. The TGF-α shedding assay was performed as previously described with a slight modification^12^. In brief, HEK 293 A cells were seeded in 6-well plates at a density of 2 × 10^5^ cells/ml. After 24 hours, the cells were transfected with a plasmid encoding AP-TGF-α (0.5 μg/well) and a plasmid encoding a GPCR (0.2 μg/well). In the screening series, eight pCAGGS plasmids encoding chimeric Gα proteins (Gαq/s, Gαq/i1, Gαq/i3, Gαq/o, Gαq/z, Gαq/12, Gαq/13, Gα16) were also transfected into the cells (0.01 μg each per well). For P2Y1, only the plasmid encoding Gαq/s was transfected (0.05 μg/well); for CB1 and CB2, only a plasmid encoding Gαq/i1 was transfected (0.05 μg/well). For P2Y4, P2Y6, P2Y10, and P2Y11, no plasmids encoding Gα proteins were introduced.

After 24 hours, the cells were suspended in Hank’s balanced salt solution with 5 mM HEPES (pH 7.4) plus Ca^2+^ and Mg^2+^. Next, the cells were mixed with either 20 μM OAD (in the screening series) or various concentrations of OAD (for P2Y1, P2Y4, P2Y6, P2Y10, P2Y11, CB1, and CB2) or its analogs (for P2Y1, P2Y4, and P2Y11), and incubated at 37 °C for 1 hour. Next, the supernatant was transferred to another 96-well plate, and 80 μL of p-nitrophenylphosphate solution^42^ was added to both supernatant and cells. Absorbance at 405 nm (OD405) was read before and after 1 or 2 hours incubation at 37 °C using a microplate reader. The following formula was used to calculate AP-TGF-α release (%):

$${\rm{AP}}\,{\rm{activity}}={\rm{\delta }}\mathrm{OD}(1\,{\rm{hour}}\mbox{--}{\rm{0}}\,\mathrm{hour})$$

$${\rm{AP}} \mbox{-} {\rm{TGF}} \mbox{-} \alpha \,{\rm{in}}\,{\rm{supernatant}}\,( \% )={\rm{AP}}\,\mathrm{supernatant}/({\rm{AP}}\,{\rm{supernatant}}+{\rm{AP}}\,{\rm{cell}})\times {\rm{100}}$$

$${\rm{AP}} \mbox{-} {\rm{TGF}} \mbox{-} \alpha \,{\rm{release}}\,( \% )={\rm{AP}} \mbox{-} {\rm{TGF}} \mbox{-} \alpha \,{\rm{mixed}}\,{\rm{in}}\,{\rm{supernatant}}\,{\rm{with}}\,{\rm{agonist}}\,( \% )-{\rm{AP}} \mbox{-} {\rm{TGF}} \mbox{-} \alpha \,{\rm{in}}\,{\rm{supernatant}}\,{\rm{mixed}}\,{\rm{with}}\,{\rm{vehicle}}\,( \% )$$

For the screening series, all GPCRs were tested in duplicate. P2Y1, P2Y10, P2Y11, CB1, and CB2 were further tested in duplicate, and P2Y6 was tested in quadruplicate.

### Statistical analysis

All statistical analyses were performed by using the Ekuseru-Toukei 2012 software program (Social Survey Research Information, Tokyo Japan). For the CB2 antagonist assay, data were analyzed by two-way ANOVA, followed by Tukey–Kramer’s test. For the assay using human PBMC-derived DCs, GPCR inhibitor assay, and P2 receptor antagonist assay, data were compared against control values (0 μM) by Dunnett’s test. For the GPCR agonist assay, data in each agonist group was compared against control values (0 μM) by Williams’ test. For the OAD analog assay, data were analyzed by Turkey–Kramer’s test for multiple comparisons.

## Supplementary information

Supplementary Table 1

## Data Availability

The datasets generated during the current study are available from the corresponding author on reasonable request.

## References

[1] Bolos M, Perea JR, Avila J (2017). Alzheimer’s disease as an inflammatory disease. Biomol Concepts..

[2] Ramirez AI (2017). The Role of Microglia in Retinal Neurodegeneration: Alzheimer’s Disease, Parkinson, and Glaucoma. Front Aging Neurosci..

[3] Chen WW, Zhang X, Huang WJ (2016). Role of neuroinflammation in neurodegenerative diseases (Review). Mol Med Rep.

[4] Salter MW, Stevens B (2017). Microglia emerge as central players in brain disease. Nat Med..

[5] G. Olmos, J. Llado, Tumor necrosis factor alpha: a link between neuroinflammation and excitotoxicity. *Mediators Inflamm*. 861231 (2014). 10.1155/2014/861231 PMC4055424 24966471

[6] Ano Y (2015). Preventive effects of a fermented dairy product against Alzheimer’s disease and identification of a novel oleamide with enhanced microglial phagocytosis and anti-inflammatory activity. PLoS One..

[7] Cravatt BF (1995). Chemical characterization of a family of brain lipids that induce sleep. Science..

[8] Leggett JD (2004). Oleamide is a selective endogenous agonist of rat and human CB1 cannabinoid receptors. Br J Pharmacol..

[9] Fernandez-Ruiz J (2007). Cannabinoid CB2 receptor: a new target for controlling neural cell survival?. Trends Pharmacol Sci..

[10] Facchinetti F, Del Giudice E, Furegato S, Passarotto M, Leon A (2003). Cannabinoids ablate release of TNFalpha in rat microglial cells stimulated with lypopolysaccharide. Glia..

[11] Oh YT (2010). Oleamide suppresses lipopolysaccharide-induced expression of iNOS and COX-2 through inhibition of NF-kappaB activation in BV2 murine microglial cells. Neurosci Lett.

[12] Inoue A (2012). TGFalpha shedding assay: an accurate and versatile method for detecting GPCR activation. Nature methods..

[13] Charlton SJ, Brown CA, Weisman GA, Turner JT, Erb L, Boarder MR (1996). PPADS and suramin as antagonists at cloned P2Y- and P2U-purinoceptors. Br J Pharmacol..

[14] Puffenbarger RA, Boothe AC, Cabral GA (2000). Cannabinoids inhibit LPS-inducible cytokine mRNA expression in rat microglial cells. Glia..

[15] Ramirez BG, Blazquez C, Gomez del Pulgar T, Guzman M, de Ceballos ML (2005). Prevention of Alzheimer’s disease pathology by cannabinoids: neuroprotection mediated by blockade of microglial activation. J Neurosci..

[16] Ashton JC, Glass M (2007). The cannabinoid CB2 receptor as a target for inflammation-dependent neurodegeneration. Current neuropharmacology..

[17] Eljaschewitsch E (2006). The endocannabinoid anandamide protects neurons during CNS inflammation by induction of MKP-1 in microglial cells. Neuron..

[18] Dhopeshwarkar A, Mackie K (2016). Functional Selectivity of CB2 Cannabinoid Receptor Ligands at a Canonical and Noncanonical Pathway. J Pharmacol Exp Ther..

[19] Kuboyama K (2011). Astrocytic P2Y(1) receptor is involved in the regulation of cytokine/chemokine transcription and cerebral damage in a rat model of cerebral ischemia. J Cereb Blood Flow Metab..

[20] Carmo MR (2014). ATP P2Y1 receptors control cognitive deficits and neurotoxicity but not glial modifications induced by brain ischemia in mice. Eur J Neurosci..

[21] Zheng W, Talley Watts L, Holstein DM, Wewer J, Lechleiter JD (2013). P2Y1R-initiated, IP3R-dependent stimulation of astrocyte mitochondrial metabolism reduces and partially reverses ischemic neuronal damage in mouse. J Cereb Blood Flow Metab..

[22] Shinozaki Y (2017). Transformation of Astrocytes to a Neuroprotective Phenotype by Microglia via P2Y1 Receptor Downregulation. Cell Rep..

[23] Fujita T, Tozaki-Saitoh H, Inoue K (2009). P2Y1 receptor signaling enhances neuroprotection by astrocytes against oxidative stress via IL-6 release in hippocampal cultures. Glia..

[24] S.J. Guzman, Z. Gerevich, P2Y Receptors in Synaptic Transmission and Plasticity: Therapeutic Potential in Cognitive Dysfunction. *Neural Plast*. 1207393 (2016). 10.1155/2016/1207393 PMC4812485 27069691

[25] Ohtani MMY, Satoh M (2000). Expression of inducible nitric oxide synthase mRNA and production of nitric oxide are induced by adenosine triphosphate in cultured rat microglia. Neuroscience Letters..

[26] Merighi S (2012). CB(2) receptors modulate ERK-1/2 kinase signalling and NO release in microglial cells stimulated with bacterial lipopolysaccharide. Br J Pharmacol..

[27] Sanz JM, Virgilio FD (2000). Kinetics and Mechanism of ATP-Dependent IL-1 Release from Microglial Cells. The Journal of Immunology..

[28] M.T. I Hide, A Inoue, *K Nakajima, *S K A.Y.N., †‡K Inoue, Extracellular ATP Triggers Tumor Necrosis Factor-a Release from Rat Microglia. *Journal of Neurochemistry*. **75**(3) (2000). 10.1046/j.1471-4159.2000.0750965.x 10936177

[29] Fiebich BL, Akter S, Akundi RS (2014). The two-hit hypothesis for neuroinflammation: role of exogenous ATP in modulating inflammation in the brain. Frontiers in cellular neuroscience..

[30] Ogata T (2003). Adenosine triphosphate inhibits cytokine release from lipopolysaccharide-activated microglia via P2y receptors. Brain Research..

[31] Boucsein C (2003). Purinergic receptors on microglial cells: functional expression in acute brain slices and modulation of microglial activationin vitro. European Journal of Neuroscience..

[32] Dreisig K, Kornum BR (2016). A critical look at the function of the P2Y11 receptor. Purinergic Signal..

[33] Costanzi S, Mamedova L, Gao ZG, Jacobson KA (2004). Architecture of P2Y nucleotide receptors: structural comparison based on sequence analysis, mutagenesis, and homology modeling. J Med Chem..

[34] Svensson M, Chen P, Hammarfjord O (2010). Dendritic Cell Regulation by Cannabinoid-Based Drugs. Pharmaceuticals (Basel)..

[35] Meis S (2010). NF546 [4,4′-(carbonylbis(imino-3,1-phenylene-carbonylimino-3,1-(4-methyl-phenylene)-car bonylimino))-bis(1,3-xylene-alpha,alpha’-diphosphonic acid) tetrasodium salt] is a non-nucleotide P2Y11 agonist and stimulates release of interleukin-8 from human monocyte-derived dendritic cells. J Pharmacol Exp Ther..

[36] Chadet S (2015). Hypoxia/Reoxygenation Inhibits P2Y11 Receptor Expression and Its Immunosuppressive Activity in Human Dendritic Cells. J Immunol..

[37] Li HQ (2013). P2Y4 receptor-mediated pinocytosis contributes to amyloid beta-induced self-uptake by microglia. Mol Cell Biol..

[38] Koizumi S (2007). UDP acting at P2Y6 receptors is a mediator of microglial phagocytosis. Nature..

[39] Tokizane K (2017). Phospholipid localization implies microglial morphology and function via Cdc42 *in vitro*. Glia..

[40] Jacobsen SE (2013). Delineation of the GPRC6A receptor signaling pathways using a mammalian cell line stably expressing the receptor. J Pharmacol Exp Ther..

[41] Jung S (2016). Conformational Constraint of the Glycerol Moiety of Lysophosphatidylserine Affords Compounds with Receptor Subtype Selectivity. J Med Chem..

[42] Uwamizu A (2015). Lysophosphatidylserine analogues differentially activate three LysoPS receptors. J. biochem..

