# Supplementary material for: Identification of P2Y receptors involved in oleamide-suppressing inflammatory responses in murine microglia and human dendritic cells
Source: Sci Rep. 2019 Feb 28;9:3135. doi: 10.1038/s41598-019-40008-8 (PMC6395661; doi:10.1038/s41598-019-40008-8)
Supplement: Supplementary file 1 — Supplementary Table 1 [file 41598_2019_40008_MOESM1_ESM.pdf]

Title:Identification of P2Y receptors involved in oleamide-suppressing inflammatory responses in murine microglia and human dendritic cells

Masahiro Kita[1*], Yasuhisa Ano[1], Asuka Inoue[2], Junken Aoki[2]

(MK and YA equally contributed to the current study.)

Supplementary Table 1. TGF-α shedding response to OAD.

Cells expressing each GPCR were treated with 20 μM OAD for 1 hour. The table presents the percentage ectodomain release of AP-TGF-α for duplicate measurements. An initial letter "m" indicates murine type; an initial letter "h" or no initial letter indicates human type.

GPCR	AP-TGFa release (%)		GPCR	AP-TGFa release (%)		GPCR	AP-TGFa release (%)		GPCR	AP-TGFa release (%)	
	#1	#2		#1	#2		#1	#2		#1	#2
GPR32	0.5	0.1	GPR50	0.2	0.6	GPR196	0.9	0.9	CRHR1	0.9	0.7
GPR38	0.7	0.7	GPR57	1.5	1.9	GPR198	0.8	0.7	CRHR2	0.3	0.9
GPR48	-0	0.2	GPR61	0.8	1.8	GPR206	0.5	0.4	VIPR2	0.4	0.1
GPR61	0.6	0.7	GPR66	0.6	0.9	GPR207	-0.4	-0	GPR44	0.8	0.5
GPR63	0.7	0.9	GPR69	1.3	1.1	GPR217	-0.2	-1	GNRHR	0.6	1.4
GPR65	1.4	1	GPR74	2.5	2	GPR218	1.7	1.9	GAVR1	0.6	0.5
GPR68	0.6	0.2	GPR76	1.2	0.5	GPR228	0.8	0.9	MCHR1	0.1	0.1
GPR82	0.5	-0	GPR84	1.5	0.7	GPR232	-0.2	-0	MCHR2	0.3	0.4
GPR87	1.2	0.7	GPR85	0.5	0.4	GPR30	0.7	0.4	GPR77	0.3	0.2
GPR88	2	0.7	GPR87	0.1	-0	GPR52	-0.8	-2	TAS2R9	-0.3	-1
GPR88	0.6	-0	GPR88	0	0.1	GPR17	-0.8	-2	TAS2R14	0.3	0.3
GPR98	1.7	0.9	GPR89	1.6	1	18B	0.4	0.9	TAS2R16	0	-0
GPR101	1.1	0.5	GPR90	1.1	1.5	CFAR1	1.4	0.5	TAAR3	-0.1	0.3
GPR107	-0	0.6	GPR94	0.4	1	GRPR	0.9	0.9	VN1R1	-0.4	-0
GPR135	-0	0.6	GPR97	0.3	0.6	CCD7	1	-0	AGTR2	-0.3	-0
GPR141	0.4	1.1	GPR99	1.8	1.7	VIPR1	0.2	-0	XCR1	-0.7	-0
GPR148	0	-0	GPR112	1.4	1	GPR104A	-0.5	-1	TAAR2	-0.6	-0
GPR150	0.2	-0	GPR117	0.1	-0	CCR1	0.2	0.5	NPY2R	0.3	-0
GPR157	-1	-2	GPR118	0.7	1.3	GPR176	0	0.5	OR1G1	0.2	0.4
GPR171	-0	0.4	GPR122	0.5	2.2	KCNJ5	0.5	0	OR3A2	0.8	0.4
AT2	0.3	0.5	GPR123	1.1	-0	RGR	0.6	1	CXCr5	0.5	0.6
GPR3	1.8	0.8	GPR126	1.3	1.4	C3AR1	0.3	-0	CCR9	0.3	0.6

GPR7	1	0.1	GPR135	0.9	0.3	OPRL1	-1.2	-0	GIT2	0.4	0.2
GPR8	0.5	0.8	GPR139	0.8	2	VIPR2	-1.2	-1	PRLHR	1.2	0.9
GPR11	0.6	-0	GPR140	1	0.9	CX3CR1	0.5	-0	GPR61	1	1.4
GPR12	1	-0	GPR142	1.2	0.4	DARC	0.1	0.5	GPR61	0.5	0
GPR14	-0	1.2	GPR143	0.3	0.4	MKNK2	0.2	-0	CCR3	-0.7	-1
GPR15	-4	0.4	GPR145	0.7	0.3	CXCR6	0.6	-0	CCR4	-0.5	-1
GPR16	0.4	0.1	GPR146	2.7	-0	CCRL1	0.5	1.9	CCr4	-1	-1
GPR17	0.7	0.1	GPR148	0.6	0.7	CXCR3	-0.3	-1	CCR8	0.1	0.1
GPR18	0.1	1	GPR149	-0.6	-1	CCR6	0.8	-0	CCR8	-0.3	-0
GPR19	1.4	0.8	GPR150	0.3	0.1	GPR182	0.5	0.5	CXCR2	-0.2	-1
GPR20	-0	-0	GPR152	0.3	0.3	LYPP1	0.3	0.4	CXCR2	0.3	0.7
GPR21	0.7	0.8	GPR158	-0.1	-0	GPRC5A	0.4	0.6	CXCR2	-1.5	-2
GPR23	0.1	-0	GPR159	1.8	1.7	GPR85	1.3	0.3	CXCR1	-0.1	-1
GPR24	1.1	0.3	GPR160	0.8	0.5	GPR173	-0.1	0.8	CXCR1	0.1	0.1
GPR25	-1	-2	GPR163	2.4	2.9	GPR77	0.8	0.6	CCR2	-6.8	-7
GPR26	-1	-1	GPR164	1	0.5	GPRC50	1.2	0.8	FSHR	0.3	0.3
GPR28	-0	-0	GPR165	1.2	0.6	GPR81	0.6	0.3	RXFP1①	0.4	-0
GPR29	0.7	-0	GPR167	0.6	0.4	GPRC5B	0.2	-0	RXFP1②	0.4	0.6
GPR31	-1	-1	GPR170	1.1	0.8	GPR61	0.2	-0	RXFP2	1.4	0.1
GPR33	-1	-0	GPR173	0.4	0.4	NMUR1	-0.1	0.1	NMBR	-1	-1
GPR37	-0	-0	GPR177	0.8	-0	HCRTR1	0.1	-0	NMBR	-1.9	-2
GPR38	1.3	0.2	GPR181	0.6	1.1	HCRTR2	0.2	0.1	BRS3	-0.2	0.6
GPR39	1.4	1	GPR184	0.2	0.5	SSTR1	0.1	0.1	GIPR	-1	1.1
GPR41	0.5	0.6	GPR189	0.1	2.3	SSTR2	-0.5	-0	GIPR	0.9	0.4

Supplementary Table 1. Continued.

GPCR	AP-TGFa release (%)		GPCR	AP-TGFa release (%)		GPCR	AP-TGFa release (%)		GPCR	AP-TGFa release (%)	
	#1	#2		#1	#2		#1	#2		#1	#2
CNR2	-0.3	-0	CX3CR1 ②	1.5	1.8	HCRTR1 ②	-0.2	-0	OPN1MW	-1.3	-1
CNR2	0.3	-0	CXCR7	0.8	0.3	MCHR1②	1.1	0.4	OPN1SW	0.1	0.8
GPR52	-0.4	0.1	GPR18②	1.3	1.5	NMUR1②	0.1	-0	OPN4	0.1	0.4

NPR2	0.7	0.2	GPR101	1.3	1.8	NMUR2	1	0.9	OR1G1②	2.5	0.6
GPR52	-0.9	-1	GPR83	1.9	1.4	NPY1R	0.1	-0	OR3A2②	-0.2	-0
GPR52	1	1.5	GALR2	0.8	1	NPY2R②	-0.2	0.1	OR51E2③	0.7	0.7
RXFP3①	0.2	-0	NPFFR1	1.9	2.5	CXCR4②	0.8	0.1	GPRC5A ②	0.2	-0
RXFP3②	0.1	-0	CCKAR ①	0.8	0.9	NPY5R	0.4	1.3	RRH	-0.6	0.5
RXFP3③	-0.1	-1	CCKBR ①	0.5	1	NTSR1	-1.6	-3	GPR173②	0.8	0.8
SSTR5	0.1	1.7	LGR5	1.3	1.4	NTSR2	-0.3	0.2	TAS2R1③	0.1	-0
TAS2R1 ①	1.4	1.7	GPR19	1.5	1.2	OPRL1	-0.6	-1	TAS2R14 ③	-0.5	0.1
TAS2R1 ②	0.4	1.2	GPR160	1.6	2.2	OPRM1	-0.1	1.1	TAS2R3②	0.5	0.2
TAS2R5 ①	-1.3	-1	TRHR①	1	0.9	PTH2R	-0.2	0.3	TAS2R5④	-0.1	0.4
CCR8④	0.3	-0	PROKR1	4.3	5.8	RAMP1	-0.8	0.5	TAS2R7	0.9	-0
TAS2R5 ③	-1.2	-1	NTSR2	5.1	4.8	RAMP3	0.3	0.7	TAS2R13 ④	0.7	0.7
CCR9②	0.1	0.1	GPR18③	1.1	1.5	GPR161	0.5	-0	EMR2	1.2	0.9
TAS2R13 ②	0.7	0.3	CXCR1②	0.5	0.8	RXFP3④	-0.2	-1	GPR62	0	0.4
TAS2R13 ③	0.4	0.3	ADCYAP 1	1.6	1.3	SSTR1②	-0.8	-1	GPR115	1.2	1.3
TAS2R10 ①	1.6	0.9	AGTR2	1.4	-0.6	SSTR2②	0.1	-1	GPR133	0.1	0.2
CXCR1③	0.2	-0	APLNR ②	0.3	0.5	SSTR3	0.2	-0	GPR142①	0.6	0.4
TAS2R7	2	0.5	C3AR1②	0.6	0.6	SCTR	-0.8	0.2	GPR142②	1	0.8
MAS1L①	0.6	0.6	GPR77②	1.1	1.2	TAAR9③	-0.1	0.4	CX3CR1②	1.3	1.9
MAS1L②	-0.7	0.4	C5AR1	2.2	2.5	TAAR8	-0.8	1.1	PTGER4	-0.6	1.5
MAS1L③	0.5	0	CALCR	0.9	0.6	TRHR②	1.2	0.6	PTGIR	1	1.5
OR51E2 ①	0.4	0.7	CALCRL	0.7	1.4	GPR81③	-0.2	0.3	GPR156	0.6	1.1
CXCR3②	0.3	-0	CCBP2	1.3	1.5	RXFP2③	0.5	-1	GPRC6A	1.2	0.7

CXCR5①	0.4	1.4	CCR1②	-0.5	0.2	PROKP2	-0.6	0.2	GRM2	0.4	0.9
VN1R1	0.8	0.2	CCR2②	1.6	0.8	TAS2R8	1	-1	OR51E1	0.5	0.7
TAS2R14 ②	1.2	0.5	CCR3②	1.4	1.4	TAS2R19	-0.1	0.7	SUCNR1	0	0.3
NPBWR2	0.7	1.4	CCR4③	1.2	0.8	CXCR5②	0.5	0	TAS2R4	0.3	0.3
GNRHR ②	1.5	0.7	CCR5	1.2	1.1	CASR	-0.6	-0	GRM8	0.1	0.6
GHSR①	2	2.3	CCR6②	-0.5	0.4	CCR2③	-1.2	-2	MAS1L②	0.2	0.8
GPR109A ②	-1.4	-1	CCKBR ②	1.7	7.3	EMR1	-1.1	-0	GPR173②	-0	-0
GPRC50 ②	-0.1	-1	CRHR1②	0.6	-0.3	GABBR1	0.6	-1	GPR135	-0.2	1.7
GPR81②	-0.4	-1	CRHR2②	-0.6	0.2	GIPR③	0.2	0.2	MLNR	0.1	0.7
RABGAP 1	0.3	-0	GPR44②	0.4	-0.2	RXFP4	2.2	1.2	OPRK1	0.6	1.4
TAAR5②	1	0.8	C9orf47 ②	0.5	1	UTS2R	-0.3	0.6	HRH1①	-0.5	0.4
TAAR9③	-0.2	0.1	GPR37	0.1	-0	GPR22	0.9	1.2	ADRA2A	-1.7	-0
TAAR6	-2.6	-1	FPR3②	0.6	0.3	GPR50	-0.2	0.2	HRH1②	-0	1
TAAR8	0.8	0.6	FSHR②	0.2	0.5	GPR52	-0.6	-0	CXCR2①	2	0.3
GPER	-0.1	-0	F2D4	-0.6	-0.1	KISSIR	0.6	0.3	GPR115	2.8	1.8
DARC	0.6	0.9	GCGR	-0.3	-0.8	TAAR2②	1.9	-0	GPR149	0.5	1.1
TAS2R8	0.4	-0	GLP2R	-0.4	-0.7	NPBWR1	1.3	1.7	OXTR	-0.3	-1
NPFFR2	-0.1	0.1	GPR173 ②	-0.1	0	GPR75	2.7	-0	PTH1R	-1.2	0.7
GHRHR	1.4	1.5	GHSR②	2.9	2.2	GPR85②	0.1	0.1	TSHR	0.4	0.7
CD97	1.6	1.7	GNRHR	-0.1	0.1	MAS1L④	-0.8	-0	VIPR2	1.1	0.4
CXCR6②	1.1	1.5	GRPR②	0.6	0.3	OPN1LW	-1	0.1	CALCR	-0.5	0.1

Supplementary Table 1. Continued.

GPCR	AP-TGFa release (%)		GPCR	AP-TGFa release (%)		GPCR	AP-TGFa release (%)		GPCR	AP-TGFa release (%)	
	#1	#2		#1	#2		#1	#2		#1	#2
GPR139	1.6	1.7	OXE	-0.3	0	B2	-0.7	0.1	mMRGA2	0.2	0.4

GPR151	0.5	1.2	P2Y1	3.9	2.7	NK1	0.6	-0	mMRGA3	1.1	1
GPR64	0.6	1.8	P2Y2	2.4	2.7	NK2	-0.2	0.2	mMRGA4	1	0.9
RXFP1①	1.2	1.6	P2Y4	0.5	2.9	NK3	0	-0	mMRGA7	3.3	2.6
BRS3	0.6	0.4	P2Y6	4.2	4	GHSR1a	2.1	3.1	mMRGB1	1.2	2.1
TAS2R13①	0.7	0.3	P2Y11	8.4	2.5	MC1R	0.4	-0	mMRGB2	3.3	1
CALCRL	0.2	0.5	P2Y12	0.2	-2	MC2R	0.3	-1	mMRGB3	0.5	0.2
CCBP2	1.6	1.6	P2Y13short	1	0.5	MC3R	-0.3	-0	mMRGB4	1.2	0.7
CCR10	0.3	1.1	P2Y13long	0.6	2.6	MC4R	0.8	-0	mMRGB5	-0.4	0.1
CCRL1	-0.2	0.6	P2Y14	2	3.5	MC5R	-0.5	0.4	mMRGB8	-0.6	-1
CXCR4①	-0.6	0.7	α1A	-0.5	0.5	MRGX1	-0.7	0.7	mMRGB13	0.4	0.7
GALR3	0.3	0.6	α1B	0	2.3	MRGX2	-0.4	0.6	mMRGD	-0.6	-1
PAFR	2	2.1	α1D	0.5	1.3	GPR119	0.8	1.5	mMRGE	1.9	2.1
CB1	0.2	0.6	α2A	1.2	0.9	OXGR1	0.2	0.7	M1	0.2	0.3
CB2	1.2	1	α2B	-0.6	1.6	GPR18	-0.3	0	MRGX1	0.1	0.4
SIP1	1.1	1.8	α2C	2.5	0.1	GPR35	3.1	3	MRGX2	-0.2	-1
SIP2	-0.6	1.4	β1	1.4	1.3	NT1R	0.2	0.1	NRGF	-0.5	-0
SIP3	3.7	2.8	β2	0.5	0.9	NT2R	1.4	1	MRGG	0.7	1.5
SIP4	0.1	0.1	β3	-0.3	0.4	GPR17short	-1	0.4	MRGH	0.8	-1
SIP5	4.4	2.9	M2	2.2	3.6	GPR17long	0.6	1.1	mLPA1	-0.5	0.5
GPR55	1.6	2.4	M3	4.7	3.6	A1	2.3	2.3	mLPA2	0.2	0
G2A	2.2	2.1	M4	1.4	3.5	A2A	0.5	0.3	mLPA3	-0.7	-1
MRGX4	1.2	1	M5	1.1	1.5	A2B	0.3	0.1	mLPA4	0.4	1.3
EP1	1.9	0.9	5-HT1A	0.9	2.6	A3	0.9	-0	mLPA5	0.6	-2
EP2	1.2	0.9	5-HT1B	-0.5	-0	D1	-1	-0	mLPA6	0.6	-0
EP3	3	1.5	5-HT1D	2	1.3	D2	-0.1	-0	mGPR34	1.4	1.6
EP3iso4	0.9	2.9	5-HT1E	0.7	1.3	D3	-1.2	-1	mP2Y10	1.8	2
EP3iso5	0.7	0.5	5-HT1F	0.7	0.5	D4	-0.8	0.5	GPR174	1.6	0.9
EP3iso6	0.9	1	5-HT2A	1.2	-0	D5	-0.1	-2	hLPA1	3.2	2.8
EP3iso7	1.6	1.8	5-HT2B	0.4	0.4	PAR1	1.7	-0	hLPA2	0.2	0
EP3iso8	0.9	0.5	5-HT2C	2.1	0.7	PAR2	0	-1	hLPA3	0	-0
EP4	0.3	1	5-HT4	0.3	1	PAR3	-0.7	-2	hLPA4	0.7	0.2

DP1	1.6	1.4	H1	1.7	2.2	PAR4	0.3	1.3	hLPA5	-0.1	0.8
TPiso1	0.5	0.5	H2	-0.7	-1	V1A	0.1	0.4	hLPA6	0	0.7
TPiso2	1.2	1.3	H3	0.5	-0	V1B	-0.3	-1	GPR34	-0.4	0.5
IP	0.3	1.4	H4	1.3	2.7	V2	-0.4	-0	hP2Y10	0.7	5
BLT1	0.3	0.5	OT	0.2	0	hMAS1	0.9	1.8	GPR174	1.3	1.1
BLT2	-0.3	0.3	AT1	0.7	0.5	hMRGD	1	0.9			
BLT2	0.6	-0	FPR1	-0.7	-0	hMRGE	0.6	-0			
CysLT1	0.3	-0	FPR2	0.9	2.6	hMRGF	-0.6	-1			
CysLT2	1.2	-1	δ	1.4	1.1	hMRGG	-0.2	-0			
FFA1	-2.1	-2	κ	-0.5	0.2	hMRGX1	-0.1	0.4			
FFA2	-0.3	-2	NOP	-0.1	0.2	hMRGX2	-0.1	0.7			
FFA3	-0.1	0.6	M	-0.1	-0	hMRGX3	0.3	0.1			
GPR120	7.2	4.8	ETA	-0.2	-1	hMRGX4	-0	2.2			
GPR84	-0.2	0.9	ETB	0	-1	mMAS1	0.4	1.1			
GPBA	0.4	0	B1	-1.1	-0	mMRGA1	0.7	-1			
